# Pregnant Females as Historical Individuals: An Insight From the Philosophy of Evo-Devo

**DOI:** 10.3389/fpsyg.2020.572106

**Published:** 2021-01-20

**Authors:** Laura Nuño de la Rosa, Mihaela Pavličev, Arantza Etxeberria

**Affiliations:** ^1^ Department of Logic and Theoretical Philosophy, Complutense University of Madrid, Ciudad Universitaria, Madrid, Spain; ^2^ Department of Theoretical Biology, University of Vienna, Vienna, Austria; ^3^ Department of Logic and Philosophy of Science, IAS Research Center for Life, Mind, and Society, University of the Basque Country, UPV/EHU, Donostia-San Sebastián, Spain

**Keywords:** evo-devo, individuality, pregnancy, reproduction, historical kinds, novelty

## Abstract

Criticisms of the “container” model of pregnancy picturing female and embryo as separate entities multiply in various philosophical and scientific contexts during the last decades. In this paper, we examine how this model underlies received views of pregnancy in evolutionary biology, in the characterization of the transition from oviparity to viviparity in mammals and in the selectionist explanations of pregnancy as an evolutionary strategy. In contrast, recent evo-devo studies on eutherian reproduction, including the role of inflammation and new maternal cell types, gather evidence in favor of considering pregnancy as an evolved relational novelty. Our thesis is that from this perspective we can identify the emergence of a new *historical* individual in evolution. In evo-devo, historical units are conceptualized as evolved entities which fulfill two main criteria, their continuous persistence and their non-exchangeability. As pregnancy can be individuated in this way, we contend that pregnant females are historical individuals. We argue that historical individuality differs from, and coexists with, other views of biological individuality as applied to pregnancy (the physiological, the evolutionary and the ecological one), but brings forward an important new insight which might help dissolve misguided conceptions.

## Introduction

The individuality of pregnancy constitutes an intriguing philosophical problem concerning the kind and number of biological individuals and the process of individuation involved. Kingma’s (2018, 2019a) metaphysical work has been pivotal for the recent philosophical reintroduction of the topic of pregnancy. Focusing on parthood relations, Kingma confronts the received view of pregnancy, where females are conceptualized as “containers” of their offspring,^1^ and argues that embryos are instead a part of a larger whole that she calls “the gravida.”

Earlier philosophical reflections on pregnancy had already criticized the container model as a view deeply entrenched both in biomedical care and everyday life, and emphasized the importance of examining the special nature of the relations between females and embryos. For instance, Young (1990) observed that pregnancy deserves phenomenological attention because it constitutes a unique way of being an individual, one involving an inner relation with another being, which is partly identical and partly extraneous to the pregnant subject. Similarly, Howes (2008) elaborated on the topic of pregnancy from an immunological perspective, and considered that both the classical container model and the understanding of the embryo as a part of “the mother’s flesh” fail to acknowledge the importance of the dynamic material *relations* between females and embryos.

As the aforementioned philosophers suggest, the prevalent biomedical conceptions of pregnancy, characterized by a consideration of female and embryo as separate biological entities, need to be reexamined. Just like insect colonies, symbiotic organisms, or the Portuguese Man-O-War, pregnancy challenges in its own way the commonsense delineation of biological entities as distinct, self-enclosed, and independent individuals. However, the problem of the individuality of pregnancy has received scarce attention within the philosophical community discussing biological individuality (but see Kingma, 2019b). The perspective we adopt in this article pays attention to recent work on the evolution of reproduction, in particular relevant evolutionary developmental biology (evo-devo) on pregnancy, to examine the philosophical question of the kind and number of individuals involved.

The field of evolution is certainly overrepresented in philosophical debates on biological individuality (Pradeu, 2016a). However, the implications of evo-devo studies for the individuation of living entities are often ignored. Even those critical of the sufficiency of evolutionary notions of individuality still tend to associate evolution with selection. In contrast with this trend, we show that extant notions of individuality do not faithfully grasp the unique biological features of pregnancy as they are highlighted in our evolutionary account, and that new criteria for historical individuation used in evo-devo render significant new insights on biological individuality.

The structure of our argument will be as follows. First, we reconstruct two main assumptions underlying the established account of pregnancy in evolutionary biology. Then, we present new studies on the evo-devo of pregnancy that show that the received understanding of reproductive modes as strategies for maximizing fitness does not suffice to explain eutherian pregnancy, insofar as it fails to consider the relational properties of reproduction and their material evolution. Thereupon, we elaborate an alternative account based on the hypothesis that pregnancy is an evolved relational novelty that gives rise to a new kind of historical individual. In the last section, we discuss how this notion differs from, but may also coexist with, other concepts of biological individuality.

## Received Views on the Evolution of Pregnancy

In this section, we provide a concise overview of the narratives that underlie classical views on pregnancy in evolutionary biology.^2^ These views have long-reaching consequences for the conceptualization of the individuals involved in pregnancy, some of which we review in this section, focusing on two main threads, namely: the emphasis on an evolutionary continuity between oviparity and viviparity, and the explanation of pregnancy as an evolutionary strategy for maximizing fitness.

Firstly, the literature on the evolution of pregnancy emphasizes a form of *evolutionary continuity* from oviparity to viviparity, in which the functions of protecting and nourishing the embryo that are fulfilled by special structures in oviparous animals (e.g., the egg shell and yolk) are transferred to the physiology of the pregnant female in viviparous animals. Accordingly, continuity is pictured as an evolutionary process of spatial internalization (Rosslenbroich, 2014). In the context of provisioning, pregnancy is regarded as a switch in patterns of embryo nutrition, from retrieving the nutrients for development from the yolk to extracting them directly from the mother *via* the placenta.^3^

Central to this narrative is the way in which the placenta, an organ of embryonic origin, has attracted enormous attention in studies of pregnancy as being the site of materialization of mother-fetus communication.

The easy accessibility of embryonal placental (in contrast to maternal uterine) tissue has likely played a major role in biasing the attention towards this organ, rather than to the uterus, as reflected by the number of scientific associations dedicated to placental research, or by the fact that there is a prominent journal devoted to it. Two major (recently revised) assumptions in evolutionary biology have further contributed to the centrality of the placenta in the conceptualization of pregnancy. One of them is the identification of the evolution of mammals with that of the placenta. In fact, the naming of Eutheria as “placental mammals” not only gives the wrong impression that the placenta is unique to eutherians, when also marsupials have one (Renfree, 2010). It also suggests that the placenta is the key innovation in the evolution of eutherian pregnancy. Altogether they seem to contend that the major evolutionary changes towards viviparity occurred solely on the embryonic side. The other assumption concerns the view that “invasive placentation” has deepened in evolution. There is a great diversity of placental types among eutherian species, with different degrees of penetration into the uterine wall; from superficial placentas, where several maternal and fetal tissue layers separate the maternal and fetal blood, to highly imbricated forms of placentation (so-called hemochorial) where fetal tissues are exposed directly to maternal blood. Since Haeckel’s times until very recently, the belief in evolutionary biology has been that early eutherian species had superficial placentas, and that “invasive placentation” is the most derived form of female-embryo interaction (see Wildman et al., 2006, for references).

Viviparity or live-bearing reproduction is a widespread reproductive mode that has arisen independently in many lineages of invertebrate as well as vertebrate animals (Wake, 2004), the latter including not only most mammals but also several clades of fishes, amphibians, and reptiles. Yet, despite a clearly eutherian-dominated view of viviparity that underestimates other forms of viviparity (Blackburn, 2015), we believe that the emphasis on the continuity between oviparity and viviparity in mammals has contributed to blurring some of the special characteristics of eutherian pregnancy. The perception of pregnancy as derived from oviparity by a simple spatial internalization followed by the gradual evolution of invasive placentation, supports an interpretation of eutherian reproduction as a mere superimposition of the embryo’s physiology on the maternal physiology, and contributes to the treatment of mother and embryo as semi-independent entities (Abbot and Rokas, 2017), and particularly to that of the pregnant female as a container to which the embryo is merely attached for nutrition.

This narrative about the phylogeny of pregnancy sets the ground for the second major component of classical evolutionary narratives of eutherian reproduction, namely the view of pregnancy as an *evolutionary strategy* involving costs and benefits for parents and offspring. In this frame, the explanation of the transition from oviparity to viviparity in mammals weighs the fitness costs and benefits of this transition for the female and her offspring, treating them as different units of selection (see Crespi and Semeniuk, 2004; Bainbridge, 2014, for reviews). In general, the internalization of development provided by viviparity is suggested to have major advantages for the offspring (such as increased survivorship by avoiding the vulnerable egg stage, increased birth size, and offspring vigor due to prolonged maternal provisioning), while entailing a mixture of advantages and costs for females. Advantages include greater mobility and smaller eggs, which are less costly to discard when unfertilized. The costs range from reduced foraging ability and higher susceptibility to predation during pregnancy, total brood loss upon death, higher energetic costs, lower fecundity, and lesser ability to interrupt the reproductive process and discard the offspring when conditions change abruptly. In sum, one should not consider that viviparity constitutes a good solution for both mothers and offspring in evolutionary adaptive terms (Avise, 2013).

The non-optimality of the “pregnancy solution” is explicit in a well-known hypothesis on the evolution of pregnancy, the so-called “conflict hypothesis”, which confronts the view of pregnancy as a “cooperative interaction between a mother and her fetus” and points instead to the potential for conflicting “interests” between maternal and fetal genes (Haig, 1993, p. 495; see also Haig, 1996). As a consequence, the genetic interests of mothers and embryos, understood as different individuals, are not perfectly aligned. The reasoning for this comes from Hamilton’s concept of *inclusive fitness*, following which the calculation of the fitness of an individual is obtained by adding the fitness contribution of relatives, weighed by the relatedness, to the direct effects on fitness. Given that mothers are likely to be more related to their further offspring than the current embryo (as current and future offspring may have different fathers), maternal investment in current pregnancy is expected to be lower than the embryo’s. According to David Haig, embryonic genes will thus be selected for gaining more nutrients from the mother, whereas maternal genes will be selected to limit that transfer. The strongest evidence in favor of genetic conflict are imprinted genes (i.e., those in which expression of alleles depends on the parent-of-origin) in the placenta. The hypothesis predicts that paternal alleles will follow the interests of the embryo, and increase maternal investment and/or prolong pregnancy, whereas the effects of maternal alleles will align with maternal interests and reduce investment. From this perspective, “the parent-offspring conflict over the degree of parental investment” is widely seen as “the main selective factor in the evolution of reproduction” (Lodé, 2012, p. 259).

All in all, the evolutionary view of pregnancy as a locus of conflict where the embryo attempts to “manipulate” the mother (see Crespi and Semeniuk, 2004) conforms with traditional approaches to the physiology of pregnancy. Biological and biomedical accounts of pregnancy often present it as a conflictual relationship between two independent entities, a “battle,” or a “combat” (Ashary et al., 2018) where the embryo uses “a variety of coercive tactics” (Ashary et al., 2018) to “manipulate” (Crespi and Semeniuk, 2004) and “invade” the mother. As a consequence, the role of the mother is often still presented as a passive or defensive one, as reflected in the biomedical depictions of the immune reaction of pregnant females upon implantation (Mor, 2007). Immune response in pregnant females would be expected for two reasons: first, because the embryo breaches physical tissue integrity during implantation, and second, because this wounding is caused by a tissue which is immunologically different from the female. However, as there is no maternal rejection of the embryo, traditional approaches have aimed to understand how the expected maternal immune reaction to implantation is “suppressed” by the fetus, for example *via* the manipulation of progesterone production, thus leading to an “immunological indolence or inertness of the mother” (Medawar, 1953; see Stadtmauer and Wagner, 2020a,b and references therein).

The explanations of pregnancy as an evolutionary strategy involving costs and benefits for parents and offspring, in continuous conflict over provisioning, and in which the female is manipulated by the embryo against her interests, reinforce the view of pregnancy as involving two separate individuals following their own interests, rather than as a reproductive process promoting constructive relations between mother and offspring. Nonetheless, this view of pregnancy as a conflict is not the only possible view of pregnancy as an evolutionary strategy. Indeed, recent models have proposed that co-adaptation (rather than conflict) between genes expressed in mother and those expressed in offspring has played a major role in the evolution of pregnancy and may offer a complementary explanation for imprinted genes (Wolf and Hager, 2006). While the treatment of maternal and offspring fitness interests in conflict theories conceives them as having separate interests, the coadaptation models assign a fitness advantage to the interaction itself, namely, to pregnancy. Interestingly, in these models the fitness interests of mothers and embryos are not only aligned, but are interdependent, i.e., fitness advantages to the mother depend on the co-evolutionary change in the fetus.

In sum, eutherian pregnancy has been studied from the perspective of there being two separate individuals, each with their own interests in evolution. As we argue in the following section, evo-devo studies of pregnancy support an alternative perspective which, instead of assuming that the results of reproduction (i.e., separate individuals) already operate in pregnancy, claims for an alternative individuation of pregnancy as the locus of developmental reproduction. In the context of the evolution of eutherian reproduction, this new kind of reproductive system constitutes what we will call a historical individual. From this perspective, it will be shown that the conflict models picturing mothers and embryos as distinct evolutionary individuals offer a partial account of the individuality of pregnancy, not only from the perspective of “proximate” disciplines such as physiology or developmental biology, but also from an evolutionary standpoint.

## Evo-Devo of Pregnancy

The way reproduction is considered in the neo-Darwinian tradition is the consequence of a long historical trajectory of work reinforcing the view that the transmission of heritable variation occurs independently of, and previously to, development. As a consequence, reproduction has been considered to consist mainly of the problem of replication, often reduced to a formal process of copy-making or a mere transmission of information (Dawkins, 1982). However, in the last decades, philosophers and evolutionary biologists have denounced that reproduction is a lot more complex than replication, as it entails the material transfer of parts from parents to offspring (Maturana and Varela, 1987; Griesemer, 2000a, 2005), as well as the reconstruction, rather than the mere transmission, of phenotypes (Jablonka, 2004; Gilbert and Epel, 2008).^4^ Therefore, reproduction and development cannot be distinguished so easily, insofar as the re-*production* of organisms is regarded as a material, organizational and developmental process, involving both the transfer of parts and the interplay of a pleiad of biotic and abiotic factors which, in the case of pregnancy, include the active role of females in the developmental reproduction of their offspring. In this sense, our view of reproduction follows many important philosophical discussions that have emphasized the importance of a developmentally minded and diachronically constructive view of ontogeny (Oyama, 2000), as well as the active role of organisms as adaptive agents in evolution (Walsh, 2015).

Despite the theoretical pleas for considering the materiality of reproduction, the evolution of modes of reproduction has remained largely unexplored so far. As Fusco and Minelli (2019) have recently denounced, “generalizations of the phenomenon of reproduction” may “have hidden the diversity of reproductive phenomena frequently found even among closely related taxa” (p. xiii). One further influencing factor for this may be that the field of evo-devo has tended to focus on the evolution of body parts rather than on the evolution of relations among organismal entities or of new kinds of biological individualities. Yet, in the last decade, studies on the “evo-devo of reproduction” have started to revert this trend. Under this perspective, modes of reproduction are not only regarded as different strategies for maximizing fitness, but also as material developmental processes involving the transformation of complex relations among organismal entities. In the remainder of this section, we present some results of recent evo-devo studies of eutherian reproduction and show how they support a conception of pregnancy that, in attributing a central importance to the evolved active maternal role and the relational novelties of pregnancy, significantly differs from the one presented in the previous section.

Recent studies emphasize that the evolution of pregnancy involved crucial innovations on the female side as a form of evolutionary reaccommodation (Stadtmauer and Wagner, 2020b). The origin of a new kind of integration between mother and embryo entailed an integral rearrangement of the interactions among the main physiological systems of the female, namely the nervous system (brain and neuroendocrine changes), the cardiovascular system (increased blood volume, decrease in hemoglobin concentration, and increased coagulation), the locomotor system (skeletomuscular changes in backbone, pelvis, and gait), and the immune and metabolic control systems (e.g., protein metabolism, and kidney capacity), to name a few (Bainbridge, 2014). All those re-accommodations involve a coevolution of extensive interdependencies between mother and offspring, both sides thus forming an evolving relational unit (e.g., Knoefler, 2010; Erlebacher, 2013; Moffet and Colucci, 2014; Pavličev et al., 2017). As we highlighted in the previous section, previous studies have abundantly focused on the evolution of the placenta. In contrast, evo-devo studies reveal that the origin of eutherian pregnancy involved crucial relational innovations on both the embryo and the maternal side. This research also counteracts the received views of pregnancy as a superficial kind of internalization in which the mother signifies a form of a living shelter for the embryo.

On the embryo side, while the placenta has originated multiple times in evolution (Renfree, 2010; Roberts et al., 2016), the kind of placentation originating in the stem lineage of *Eutheria* is unique, in particular with regard to the degree of maternal-fetal integration it confers (Wildman et al., 2006). Eutherian placentation breaches maternal integrity and is associated with implantation. In stark contrast to non-mammalian viviparous animals in which the placenta is only apposed to the uterine epithelium, the maternal-placental interface of eutherian mammals erodes the uterine epithelium or even the maternal vessel walls. As we saw in the previous section, the received assumption on the evolution of the placenta was that invasive placentation evolved from superficial placentas with a shallow contact between the maternal and the embryonic tissues. In contrast, phylogenetic analyses have recently shown that the invasive placental type was indeed the ancestral state of all eutherians, indicating that eutherian pregnancy arose concomitantly with the origin of a highly entangled maternal-fetal interface (Mess and Carter, 2006; Wildman et al., 2006).

On the maternal side, recent research has revealed that new specialized cell types, such as the decidual stromal cell, the uterine natural killer cell, and a specialized form of resident macrophages, evolved likely coincidentally with the evolution of pregnancy (Wagner et al., 2014; Erkenbrack et al., 2018). Particularly interesting is the decidual stromal cell type, which evolved together with invasive placentation (Chavan et al., 2016; Erkenbrack et al., 2018). These maternal novelties likely enabled sustained implantation and therefore the evolution of the first step towards eutherian pregnancy. Just like in the case of the placenta, the novelty of the uterine cells relies not only on their inherent characteristics, but on their relational abilities, that is, on their capacities to communicate with other (in this case, genetically heterogeneous) cells (see Griffith and Wagner, 2017). Indeed, impaired decidualization of endometrium has been shown to interfere with embryo-maternal interactions in humans, thus causing recurrent pregnancy loss (Salker et al., 2010).

**Figure 1:**
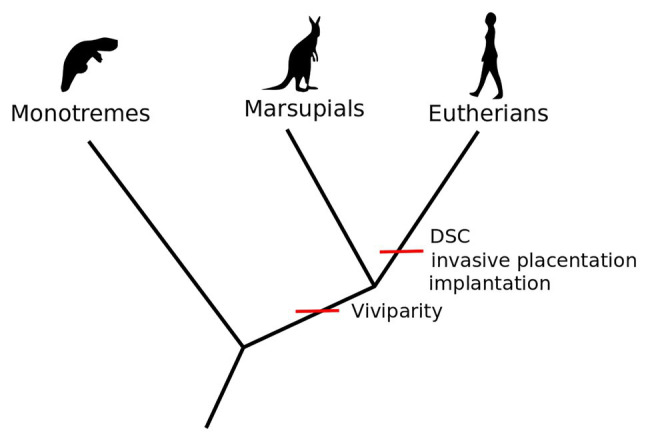
Viviparity is a shared derived trait of marsupials and eutherians. Embryo implantation, invasive placentation, and decidual stromal cells (DSC) occur only in the eutherian lineage [Adapted from Wagner et al. (2019), Figure 1, p. 2].

Crucial to this new understanding of the relational novelties emerging in eutherian reproduction have been the studies on the role of inflammation in the origination and prolongation of pregnancy. Pregnancy has been traditionally described by reproductive biologists as a period between two discrete events, implantation and birth, both of which have been shown to entail inflammation (Mor, 2007; Mor and Cardenas, 2010; Mor et al., 2011). Whereas in marsupials the inflammation caused by the first contact of the fertilized egg is followed by expulsion (birth), and thus the period of pregnancy is very short, in eutherian mammals inflammation is a required step for successful implantation and does not result in immediate birth. In eutherian pregnancy, the inflammatory response is thus modified by the maternal decidual cells to separate inflammatory implantation from expulsion (Chavan et al., 2016; Griffith et al., 2017). Thus, the maternal immune system is not simply suppressed. Rather, the evolution of decidual cells enabled its temporally and spatially local modification, making implantation possible (Mor and Cardenas, 2010; Mor et al., 2017)^5^ and subsequently expanding pregnancy and maintaining an alternative stable homeostatic state. This sequence of events in eutherians evolved after the last common ancestor with marsupials, who do not have decidual cells and react to attachment with expulsion. The eutherian novelty hence consists of the novel cell type enabling a prolonged intrauterine developmental stage to be “inserted” between two inflammatory events, namely implantation and birth (Griffith et al., 2017; Erkenbrack et al., 2018; see Figure 1).

In the next section, we present our main claim that the evolutionary modifications that led to the origination of pregnancy (female integral reaccomodation, emergence of a new type of placentation and uterine cell type, and modification and repurposing of inflammation) may be interpreted as a transition in individuality in which two individual processes, the adult female and the developing embryo, are *merged* into a single reproductive individual of a historical kind.

## Pregnant Females as Historical Individuals

The features of the evolution of eutherian reproduction as reviewed in the previous section prompt us to propose that pregnant females constitute a new kind of individual appearing in evolution. In this section, we examine some of these features in the light of conceptual work on historical kinds developed in the field of evo-devo, and argue that pregnant females can be considered to be biological individuals of this historical kind. The notion of historical kind has been characterized as including “a subset of natural kinds that acquires, through evolutionary processes, a quasi independent lineage-history” (Wagner and Tomlinson, 2020, p. 1). Historical kinds “have a definite beginning and potentially an end” (Wagner, 2001, p. 10) and, therefore, allow to combine in the same concept, as two sides of the same coin, the evolutionary origination of new processes, structures and functions, and their historical persistence throughout evolutionary time.

Understanding individuality as a historical kind encompasses a set of criteria for individuation of evolutionary entities used in the context of evo-devo. The criteria used in this field to track the historical emergence and persistence of entities such as homologues and body plans, differ from the traditional criteria for evolutionary individuation, and enable evo-devo biologists to individuate evolutionary units in distinct ways. Evolutionary entities in evo-devo have been mainly conceptualized as types or natural kinds (see Wagner, 1996; Brigandt, 2017, for a review), and here we propose to extend this view to kinds of individuals. While this perspective has classically been applied to the individuation of body parts, such as vertebrate limbs or cell types, it has also been extended to include developmental stages (e.g., larval vs. adult stage), physiological processes (e.g., menstruation or ovulation), or functions (e.g., behaviors; see, e.g., Gilbert and Bolker, 2001; Scholtz, 2005; Love, 2007). We argue that the criteria for historical individuation can be further applied to entities arising in reproductive relations, and enable a view of the pregnant female as a new kind of individual, namely a historically new, semi-independently modifiable developmental stage in the life cycle of (some) eutherian females, with continuous persistence since its origination.

### Criteria for Historical Kinds

Historical units are evolved entities or processes which fulfill certain criteria that allow us to recognize them as distinct, namely, their continuous persistence across taxa and throughout evolutionary time, and their non-exchangeability with other such units. As we will see, pregnancy can be inviduated in this way because it fulfills these two criteria.

The first criterion to track historical individuals, *persistence*, does not derive from the direct replication of an entity (such as a limb or a cell), but rather from those developmental processes that account for the historical continuity of an entity within and across species. As a consequence of their developmental autonomy, these entities can change or remain stable throughout evolution somewhat independently from others (Wagner and Altenberg, 1996). The classical example is the vertebrate limb, which adopts different shapes and sizes across vertebrates, adapted to different functions, but it yet persists as a distinct, developmentally grounded, historical kind.

The pregnant female as a historical individual evokes an evolutionarily persistent entity in which female and embryo are developmentally entangled. This is manifest in the form of a transient, but temporally demarcated, individuality characterized by a high degree of integration between female and embryo. As argued in the previous section, the origination of pregnancy entailed a major modification of the relational abilities of mammalian females, one that allowed pregnant females to internalize embryos as parts of a new reproductive system. The inflammatory events following implantation and preceding birth individuate pregnancy in time: both the onset and finalization of pregnancy are coordinated relational events between mother and embryo, rather than occurring when the embryo one-sidedly reaches certain stages of development or maturation. In this frame, reproduction is thus treated less as a point event in the lifetime marked by fertilization, and more as being itself a developmental process. This diachronic view of historical individuality as applied to the reproductive phase of pregnancy aligns, as suggested to us by an anonymous reviewer, with recent work on the biology of reproduction (Fusco and Minelli, 2019), where biological individuality is drafted within the framework of life cycle evolution (DiFrisco and Mossio, 2021).

Moreover, the persistence and distinctiveness of historical individuals are not only reflected in their evolutionary continuity but also in their distinctive ability to evolve. Therefore, as a consequence of individuation, eutherian pregnancy obtains a certain degree of evolvability on its own, insofar as it inaugurates new ways of generating variation and therefore new potential to evolve. The relative ability of the pregnant female to evolve as a unit is reflected, for example, in the variability of eutherian species in the length of gestation, or in the characteristic diversification of the maternal-placental interface (Carter and Enders, 2004).

The second criterion for historical individuality, *non-exchangeability*, captures the idea that the evolutionary autonomy of a new historical entity does not result from the disconnection of this entity from others, but rather from an evolutionary process of compensation and accommodation of developmental and physiological interdependencies within the organization of a body plan, thus resulting in a new kind of evolved integration. For example, if vertebrate hind limbs can be individuated as historical individuals it is not only because they change independently of forelimbs (and of everything else), but because they are non-exchangeable. The reason is that, although they develop using some of the same genes and developmental pathways, hindlimbs are different (and evolve differently) from forelimbs also due to their integration in the distal part of the vertebrate body. In contrast, human hairs cannot be considered as historical individuals: while they are physically independent entities, they are “exchangeable” in the sense that the identity of each hair does not depend on their particular location in the skin. The distinctiveness of historical kinds is thus based both in their evolutionary autonomy and in their evolved integration within the system they belong to.

From this perspective, the mode of evolution instantiated by the integral reaccommodation of all the physiological systems that make up eutherian reproduction (including the embryo) is not surprising. Evolution is a process in which new traits and relations emerge not by mere addition of new developmental stages or structures on top of the preexisting, conserved ones, but by the recruitment, modification and integration of the old into a new context (Alberch, 1985). Classic models in vertebrate evo-devo include studies on the origin and evolution of pharyngeal jaws, which involved the integration of changes in the visual, neural, skeletal, muscular, and behavioral systems. In the words of Brian Hall, “[s]uch studies move us away from identification of single key innovations and toward an emphasis on integrated changes and ontogenetic repatteming in interrelated systems” (Hall, 1998, p. 282). As we saw in the previous section, the novelty of pregnancy not only entailed the emergence of new relational structures, processes, and functions, but also the modification of a range of pre-existing physiological self-maintaining systems to support a distinctly new homeostatic state that incorporates the implanted embryo (Pavličev et al., 2017). Therefore, the individuation of pregnancy does not occur by decoupling pregnancy from the rest of female biology, but rather by the unique modifications of female physiology (i.e., capacity for changes in immune, metabolic, and locomotory systems) that enable pregnancy and hence integrate it with other developmental stages in its life cycle. This integration includes the accommodation of the embryo, as reflected in the evolution of the female immune system. In general, two evolutionary “solutions” to a conflicting situation (such as that triggered by the disruption of tissue integrity caused by embryo implantation) could be considered. One might consist of removing the origin of the conflict entirely, and the other of integrating and modifying it.^6^ In contrast with the received understanding of pregnancy as an ongoing conflict, evo-devo studies of the origin of pregnancy suggest that implantation leads to a critical disruption of physiological homeostasis (Erkenbrack et al., 2018), followed by its overcoming, which results in a novel homeostatic state defined at the relational level. It is this new function and the associated developmental and physiological processes that evo-devo studies of eutherian reproduction aim at explaining.

### The Origin of Pregnant Females as Historical Individuals

In contrast with the most prominent work from the neo-Darwinian perspective on pregnancy, evo-devo studies of eutherian reproduction concern the evolutionary origination, rather than the modification, of pregnancy. In this section, we argue that the kind of transformations involved in this transition is not simply assimilable to an evolutionary novelty with an associated new function, as in the origin of characters such as feathers or paired fins. Rather, the origin of pregnancy has meaningful correspondences with major transitions such as the origin of eukaryotic cells or multicellulars, which often entail new modes of reproduction (Griesemer, 2000b) and the emergence of new levels of evolutionary individuality (Buss, 1987; Michod, 2000).

On a first glance, the case of pregnancy does not seem to fit in the standard view of major transitions (Maynard Smith and Szathmary, 1997): unlike eukaryotic cells or multicellular organisms, pregnant females certainly do not reproduce directly into pregnant females. However, the systemic transformations and the radical changes in reproductive capacities experienced by eutherian females indicate that the origin of pregnancy had further evolutionary implications than that of a new reproductive character. In particular, the origin of eutherian reproduction did entail that “entities that were capable of independent reproduction before the transition, can reproduce only as parts of a larger whole after it” (Griesemer, 2000b, p. 79). In this sense, the transition to pregnancy might be considered as analogous to the transition to the eukaryotic cell, described by Godfrey-Smith (2015, p. 10123) as the event in which “two simple reproducers give rise to collective reproduction, followed by a loss of reproductive autonomy and the endosymbiont moving towards scaffolded reproduction.” In an analogous way, eutherian pregnancy entailed a loss of reproductive autonomy at the level of the egg, but a gain of reproductive capacity at the new individual level constituted by the pregnant female. In this sense, pregnancy can be considered as a last of the successive evolutionary stages of female integration of reproduction: from releasing an unfertilized egg to be fertilized and developed externally, to internal fertilization followed by a largely external development (i.e., oviparity), to metatherian viviparity, in which case both fertilization as well as great part of development are incorporated within the female’s body. This integration importantly varies in extent and time: in some mammalian species, development has evolved to become integrated with reproduction until a certain stage (marsupials, those with an extremely short gestation period), while the extension of pregnancy has allowed eutherians to integrate development and reproduction until a much later stage. In eutherians, development and reproduction have become highly integrated processes, insofar as the reproducing individual (the pregnant female) needs to participate in the development of its offspring to achieve its own reproduction. To sum up: pregnant females form unique individuals, relating two developmental processes at different stages of their life histories. They are reproductive, relational, and transient individuals, although, like most biological individuals, they have a beginning and an end: they are born at implantation and end at birth.

In philosophical terms, the concept of historical individual as applied to pregnant females delivers a new insight to the notion of biological individual, one which is distinctly evolutionary and which differs from the conflict models. As pointed out in the introduction, philosophical debates on biological individuality have too often been posed in evolutionary terms to the detriment of other biological fields (Pradeu, 2016a, p. 765). However, it is important to stress that the implications for individuality of non-selectionist, developmental approaches to evolution have been also neglected. The thesis that pregnant females are historical novel individuals relies on an evolutionary stance, yet it is a very different one with regard to previous selectionist accounts. In the following section, the main concepts of individuality discussed in the philosophy of biology are reviewed and compared according to their adequacy to account for pregnancy in contrast to the historical notion advanced here.

## Pregnancy and Biological Individuality

The nature of biological individuality has been a topic of intense inquiry in the philosophy of biology of the last decade (Ruiz-Mirazo et al., 2000; Clarke, 2010; Pradeu, 2016a; DiFrisco, 2019), where received assumptions have been revised to respond to new challenges coming from entities that do not conform to traditional concepts of individuals considered as homogeneous, unique and functionally integrated entities (Santelices, 1999). Insofar as reproduction is generally regarded as the process by which new individuals are generated, the notion of individuality plays an inevitable central role in studies on reproduction (Fusco and Minelli, 2019, p. 25). However, despite this apparent centrality of individuality in reproduction, pregnancy has not received much attention in the context of this debate. Recently, Kingma (2019b) has tentatively discussed how some criteria for biological individuality (taken from Clarke, 2010) may apply to the entities involved in mammalian pregnancy. Kingma does not defend these criteria or their application, but poses “[t]he merit of the exercise in raising the question.” In contrast, in this paper, we do take a stance for a given understanding of biological individuality in the case of pregnancy. In this section, we contrast our proposal of pregnancy as a historical kind of individual with the three core concepts of biological individuality currently discussed in the philosophy of biology, namely the physiological, the evolutionary, and the ecological approaches, and consider their merits and shortcomings as applied to the individuality of eutherian pregnancy (see Table 1).

**Table 1:** Comparative table of concepts of biological individuality and how they apply to pregnancy.

	Entities	Criteria for individuation	Number and kinds of individuals in eutherian pregnancy
Physiological	Organisms	Self-maintenance Functional integration	One (Part-whole) Two (Container model)
Evolutionary	Genes Organisms, Species	Units of selection	Three (mother’s, father’s, and embryo’s genes)
Ecological	Ecological networks	Interdependence Scaffolding	One (holobiont approach) Two (scaffolding approach)
Historical	Phenotypes (body parts, developmental processes, physiological functions)	Historical persistence Non-exchangeability	One (reproductive system)

### Physiological Individuality

The physiological notion of individuality captures the most intuitive view of biological individuals as autonomous, functionally integrated, and self-maintaining systems, separated from their environments. It underlies the classical views of “organisms” developed by the physiological tradition in biomedicine (e.g., Perlman, 2000), as well as the organizational approach in contemporary philosophy of biology (Ruiz-Mirazo et al., 2000; Moreno and Mossio, 2015). Criteria for physiological individuation comprehend how different functionalities contribute to self-maintenance. More recently, they have been expanded to include how immune mechanisms enable the delineation and persistence of physiological individuals (Pradeu, 2010, 2016b).

From the physiological perspective that guides biomedical and bioethical approaches to human pregnancy, it is generally considered that pregnancy encompasses two separate organisms, namely, the mother and the embryo. While the status of mothers as physiological individuals is generally seen as trivially uncontroversial, there is no consensus concerning the stage at which embryos begin to have a separate individual existence in development. Different developmental events have been proposed to mark the transition to physiological individuality in human embryos, including fertilization (Damschen et al., 2006), implantation (Alvargonzález, 2016), gastrulation (Smith and Brogaard, 2003), or completion of organogenesis (Nuño de la Rosa, 2010). In contrast, recent contributions have challenged the assumption that females preserve a physiological individuality independent of their offspring during pregnancy. As mentioned before, Howes (2008) concluded that immune interactions blur the traditional boundaries assumed between mother and offspring, and offered a third relational, “not-one-but-not-two,” alternative emphasizing the dynamic physical interactions between female and embryo. More recently, Kingma (2018, 2019b) has argued that, until birth, fetuses do not fulfill the traditional criteria for biological individuality, such as being bounded by topological frontiers or delineated by physiological or immunological mechanisms. Instead, she suggests that it is pregnant females, inclusive of their fetuses, that should be considered as individuals, although she admits her position to be compatible with the possibility that fetuses are also individuals.

Kingma’s mereological approach to the metaphysical status of pregnancy illustrates a general trend in debates on “organismality”, which, in focusing on criteria for delineating the spatial identity of organisms (i.e., “which sorts of parts should be included within the spatial boundaries of individuals”), have tended to neglect the problem of the diachronic identity of organisms (i.e., “which sorts of events should be included within the temporal boundaries of a life”; DiFrisco and Mossio, 2021, p. 177). In contrast, the inflammatory events associated with implantation and birth provide diachronic criteria for the individuation of pregnancy, which, in turn, can be characterized by the specific series of developmental events constituting this developmental stage.

In this sense, pregnant females might not be best viewed as being themselves organisms, but rather as developmental stages in the life cycle of certain (eutherian) organisms. After all, life cycles of most plant and animal groups involve dramatic developmental transformations and varied reproductive phases (Fusco and Minelli, 2019). Just like metamorphosis, pregnancy might be considered as a new organizational form associated with a new developmental stage, rather than as a new individual. However, we believe that the spatio-temporal criteria for physiological individuality do not exhaust the kind of individuality that pregnancy brings about. Besides that, pregnancy needs to be recognized as a *reproductive* individuality which is irreducible to that of developmental or physiological individuality. Unlike the physiological systems participating in organismic maintenance (such as the digestive, circulatory or respiratory systems), reproductive functionalities are not just contributions to the self- or the scaffolded homeostasis of individual organisms, but to a different type of homeostasis, namely the maintenance of pregnancy as a relational process that might involve different physiological individuals (Pavličev et al., 2017; Stadtmauer and Wagner, 2020a,b). As a consequence, physiological and reproductive criteria of individuality do not necessarily render the same entities, although they might overlap at certain stages of the life cycle. Thus, embryos might be considered to be physiologically individuated before birth, but to belong to the reproductive system until birth. In this sense, even if birth is seen as an arbitrary event from the perspective of the physiological individuality of embryos, it sets a temporal limit to reproductive individuality, insofar as it breaks the relation inaugurated by implantation and entails an integral reaccomodation of both the female and the embryo physiologies.^7^ It is this new kind of reproductive individual, we claim, that is individuated in evolution, giving rise to a novel historical individual which includes the whole lineage of eutherian pregnant females.

### Evolutionary Individuality

The evolutionary notion of individuality sets the mainstream view in the philosophy of biology. In the conceptual framework of the Modern Synthesis, individuals are understood as theoretical entities of evolutionary biology, namely, those that play a role in the theory of evolution by natural selection, their main features being variation, heritability, and differential fitness (Godfrey-Smith, 2013). From this perspective, entities below and above the level of the organism, such as genes, groups or species, can also work as individuals understood as units of selection.

As we saw above, the conflict hypothesis is the mainstream hypothesis in evolutionary explanations of pregnancy. This view attributes interests to the genes (alleles of maternal and paternal origin), which are “expressed” through their interactors: mothers and embryos, the latter acting as the vehicle of both paternal and maternal interests. Although conflict applies to the genes and not to their carriers, as Haig (2014) himself has warned about, under this model, pregnancy features as a place of negotiation of the presumed interests of separate individuals (namely, the mother, the father, and the embryo), rather than as a biological system on its own. In contrast, our notion of historical individuality reveals an important contrast to this conventional evolutionary conceptualization of pregnancy. While, from an evolutionary genetic perspective (leaving mitochondrial genes aside), paternal and maternal roles are ontologically equivalent, from a reproductive perspective, they are not. The reason is that the latter account integrates into the process of reproduction the genetic, morphological, developmental, and physiological processes which affect material reproductive relations among living systems and which result in the production of a new organism with a new life history.

Nonetheless, evolutionary approaches to individuality are not necessarily committed to a gene-centered view of reproduction. Under non-reductionist approaches to Darwinian individuality where organisms, groups, or even species can be considered as units of selection, pregnant females including their offspring might be seen as evolutionary individuals seeking to maximize fitness. According to the criteria used by Clarke (2010) or Godfrey-Smith (2013), pregnant females would not be considered as single evolutionary individuals because mother and offspring are genetically different, even though they have partially overlapping fitness interests. Nonetheless, Kingma (2019b) seems to reach the opposite conclusion when she analyzes the individuality of pregnancy from an evolutionary perspective. In this case, it might be argued that our proposed notion of historical individual and that of evolutionary individuals overlap for the case of pregnancy, thus rendering ours superfluous. However, we believe that the virtues of identifying new kinds of biological individuals do not lie in their distinctive delineating capacities, but rather in their abilities to explain phenomena that other notions of individuality are unable to explain (DiFrisco, 2019). Tracking the pregnant female as a historical individual accounts for the developmental basis that explains the boundaries and persistence of pregnancy, the distinct evolvability of this reproductive system, and the associated changes that take place in the eutherian lineage after the emergence of pregnancy. None of these phenomena belongs to the *explananda* of selectionist explanations of pregnancy as a reproductive strategy.

### Ecological Individuality

An important contribution to the debate on biological individuality has surfaced in the last decade out of the greater attention paid to how relations of organisms with the biotic and abiotic milieu challenge some of our received assumptions on individuality. While the ecological notion of individuality (Huneman, 2014) can be applied to composites including nonliving parts, it has been particularly influential in discussions on the status of multi-species partnerships (Queller and Strassmann, 2016; Hernández and Vecchi, 2019), and more specifically of symbiotic associations (Gilbert et al., 2012; Gilbert and Tauber, 2016). So-called “holobionts” challenge the view of individuals as non-problematic well-bounded entities, some claiming that certain symbiotic associations can be understood as collective individuals (Chiu and Gilbert, 2015) or as “hybrids” made of individuals of different lineages (Chiu and Eberl, 2016). Importantly, ecological reflections on the status of symbionts do not necessarily attempt to replace the physiological and evolutionary criteria of individuality. Rather, symbionts might be individuated differently depending on the adopted perspective. Thus, some consider holobionts as units of selection (Roughgarden et al., 2018), while others admit that some symbionts do count as physiological, but not as evolutionary, individuals (Godfrey-Smith, 2015).

Debates on the consequences of symbiotic relationships for the individuation of biological entities have an obvious counterpart in thinking of the individuality of pregnancy. One might claim that females and embryos are contingently engaged forming a heterogeneous entity, whereas from the holobiont perspective, one could posit that the pregnant female is a collective individual including female and embryo(s) as same species parts, together with allospecies microbiota. This is the line followed by Chiu and Gilbert (2015) when they argue that the interactions between mother, fetus, and symbionts during pregnancy reciprocally construct each other’s experienced environments, facilitating the scaffolding of their development and reproduction.

Scaffolding has been a candidate model for understanding the pregnancy relation. The notion of scaffolding has been conceived of in manifold ways. Sometimes scaffolds are defined as those organic resources used in development and reproduction, that, contrary to those fueling metabolism, are not incorporated into the system (Minelli, 2016). These include parents, members of symbiosis, and non-living products of metabolism. In sum, resources that are required to explain, yet remain distinct from the scaffolded organism. In this context, pregnancy has been conceptualized as a source of nutrition for the embryo. In other cases, developmental scaffolding is interpreted as an instance of the evolutionary tendency towards exploiting increasingly organized developmental environments (Griesemer, 2014a). Then pregnancy appears as providing a new “ontogenetic niche” (i.e., the uterus; Stotz, 2008) that increases the reliability of development (Chiu and Gilbert, 2015). Following a further relational insight, scaffolding may include not only interactions between developers and scaffolds, but also “prostheses,” i.e., those parts that, like nests, enhance or substitute for developed parts (Griesemer, 2014a). Under this view, pregnancy might be seen as one of many possible parent-offspring relations, in which some form of strong collaboration transiently emerges. For instance, Griesemer discusses the example of haptic contact between a parent and her child holding hands to cross a street as a form of hybrid individual characterized by their temporary fusion (Griesemer, 2018).

Yet, we believe that in eutherian reproduction, the female is not a mere scaffold for embryonic development, either conceptualized as a stable environment, a source of nutrition, or a facilitator of development. Rather, mother and embryo participate in the *co-production* of the offspring, by forming a transient reproductive individual. In our view, the distinct status of pregnant females as compared to other forms of ecological individuality resulting from scaffolding relationships lies in the historical, intrinsic nature of the relation, in that it is itself an evolved entity, in which both sides of the relation are modified specifically in, and for this relation. In the case of pregnancy, its individuality is transitional, but it does have a beginning and an end: pregnancy inaugurates a reproductive individual in which female and embryo are transiently entangled from implantation to birth. This does not mean that pregnant females are the only instance of historical individuals including heterogeneous entities. Some kin associations such as insect colonies (which also include members of the same species at different stages of their life cycle) or multispecies aggregates such as symbiotic ones might be considered as historical individuals in a similar sense.

Our brief survey of the main current notions of biological individuality and the stance(s) of each in the case of pregnancy, confirms the current pluralist consensus on the topic (Pradeu, 2016a). Most participants in the debate agree that different notions of biological individuality depend on the questions asked or the perspective favored for solving a particular problem, and are largely relative to the methods and practices used to individuate empirical processes of concern in each disciplinary context (Bueno et al., 2018; Griesemer, 2018; Love, 2018). We have shown how different approaches to individuality, as inspired in the epistemic goals of different biological disciplines, use non-overlapping criteria of individuation that lead to different delineations and conceptualizations of pregnancy. More importantly, in looking at practices of individuation in evo-devo, a neglected field in the philosophical debate on individuality, we have identified a new concept of biological individuality. As applied to pregnancy, our concept of historical individuality, according to which pregnant females are evolved forms of individual living organizations, brings forward a new perspective not covered by the rest of the conceptions.

## Conclusion

The main aim of this article has been to challenge the received view of pregnancy as consisting of two separate individuals, and to offer an alternative conception stemming from recent evolutionary developmental studies. Thus, we have argued that eutherian reproduction is characterized by a developmental integration of physiological and immune processes so that pregnant females need to be accounted for as individuals. We have proposed a novel notion of biological individuality to account for this, namely that of *historical individuality*, according to which living entities, including pregnant females, are individuated using the evo-devo criteria of persistence and non-exchangeability. The individuality of eutherian pregnancy is of a historical reproductive kind because it originated in evolution as a particular organization of relations that fulfill those criteria.

Concepts of individuality are required “in order to tell stories about what goes on in the world, do science, and make attributions of properties, relations, responsibility (causal or moral), and standing (e.g., epistemic, moral, and legal).” (Griesemer, 2018, p. 138). Although we do not deal with this issue in this paper, it is evident that both biology and medicine have so far overlooked the individuality of pregnant females, and this has had far-reaching consequences, not only for biomedical practices on human pregnancy, but also for social interpretations of reproduction. We think that taking into account in those fields the thesis we present here, namely, that pregnant females are historical kinds of individuals, can positively contribute to reverse important misconceptions.

## Author Contributions

All authors listed have made a substantial, direct and intellectual contribution to the work and approved it for publication.

### Conflict of Interest

The authors declare that the research was conducted in the absence of any commercial or financial relationships that could be construed as a potential conflict of interest.The handling editor declared a shared research project with one of the authors LN at time of review.

## Footnotes

^1^Following Blackburn and Starck (2015) we use the term “embryo” in a broad sense (from implantation to birth) that also includes fetuses.

^2^Social representations of scientific knowledge use metaphors influenced by social stereotypes (see e.g., Wagner et al., 1995 for the topic of conception), but also scientific accounts are influenced by social, and particularly by gender biases (see Martin, 1991, for the same topic). The language used in biological accounts of pregnancy is a particularly good illustration of how the social perception of a biological process has influenced its scientific interpretation, and vice versa.

^3^The placenta evolved from the vascularized membranes of the yolk sac and/or allantois, which in oviparous animals supply nutrients from yolk and gas exchange with the external environment. In contrast, in viviparous mammals the membranes fuse with the chorion to form the placenta, which attaches to the uterine wall and serves as continuous mediator of nutrients and gas exchange with the maternal blood (Ramsey, 1982; Carter, 2012).

^4^While there have been recent attempts to improve the understanding of reproduction from a Darwinian perspective, they still regard eutherian reproduction essentially as the capacity to make reliable copies of an individual entity. For instance, Godfrey-Smith’s (2015) recent distinction of different forms of reproduction classifies eutherian mammals as “collective” reproducers which, just like any other multicellular, have parts with the capacity to reproduce, while viruses are seen as “scaffolded” reproducers whose reproduction depends on external resources. In contrast, from the reproducers perspective, almost all cases of reproduction are seen as scaffolded (Griesemer, 2014a,,b; Minelli, 2016).

^5^It is important to note that the modification of the immune reaction must be local, not system-wide or persistent, because it would otherwise likely be lethal for the mother.

^6^Wagner and co-authors go a step further to suggest that stress pathway-inducing processes offer a particularly strong opportunity to generate novelties, by first internalizing, and then modifying an originally plastic stress response (Erkenbrack et al., 2018; Wagner et al., 2019).

^7^It might be argued that birth is an arbitrary event from a reproductive perspective, given the extended period of neonatal immaturity that follows birth in some mammals, and their dependence on lactation. In this view, the physiological individuality of pregnant females would gradually disintegrate after birth by changing the set of maternal-offspring interdependencies to others, individuality becoming a matter of degree. While this is a plausible interpretation, such a position would not be able to individuate pregnancy, but rather the more general kind of relationship mediating mothers and offspring among mammals, including oviparous mammals.
